# Emerging Roles for Eph Receptors and Ephrin Ligands in Immunity

**DOI:** 10.3389/fimmu.2019.01473

**Published:** 2019-07-04

**Authors:** Thayer K. Darling, Tracey J. Lamb

**Affiliations:** ^1^Immunology and Molecular Pathogenesis Program, Emory University Laney Graduate School, Atlanta, GA, United States; ^2^Department of Pathology, University of Utah, Salt Lake City, UT, United States

**Keywords:** Eph, ephrin, activation, cell trafficking, migration, adhesion, inflammation, disease

## Abstract

Eph receptors are the largest family of receptor tyrosine kinases and mediate a myriad of essential processes in humans from embryonic development to adult tissue homeostasis through interactions with membrane-bound ephrin ligands. The ubiquitous expression of Eph receptors and ephrin ligands among the cellular players of the immune system underscores the importance of these molecules in orchestrating an optimal immune response. This review provides an overview of the various roles of Eph receptors and ephrin ligands in immune cell development, activation, and migration. We also discuss the role of Eph receptors in disease pathogenesis as well as the implications of Eph receptors as future immunotherapy targets. Given the diverse and critical roles of Eph receptors and ephrin ligands throughout the immune system during both resting and activated states, this review aims to highlight the critical yet underappreciated roles of this family of signaling molecules in the immune system.

## Eph Receptors: a Paradoxical Family of Kinases

**Figure 1:**
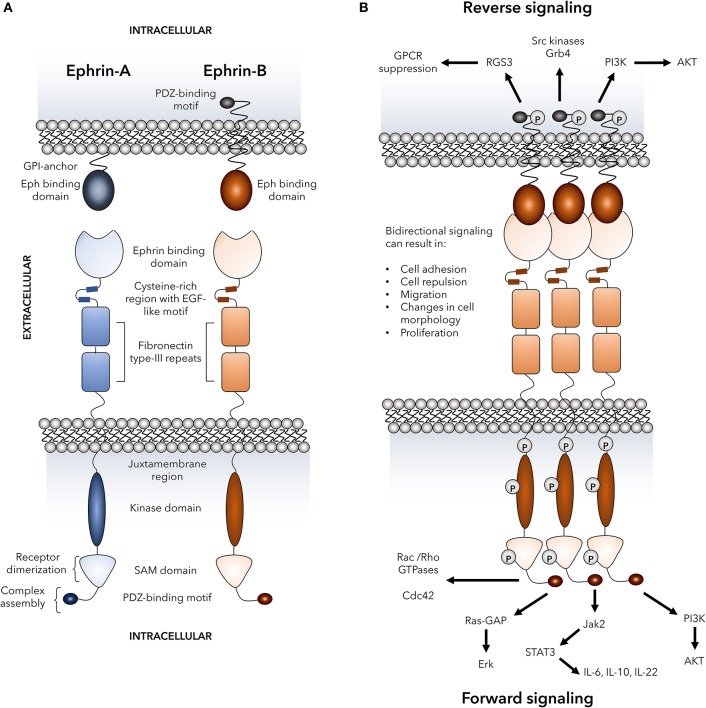
Basic Eph receptor structure and signaling pathways. The structure of Eph receptors and their ligands is shown in **(A)**. Eph receptors are consisting of an extracellular structure consisting of an ephrin binding domain connected to two fibronectin type-III repeats by a cysteine-rich EGF-like motif. The juxtamembrane region connects the extracellular portion of the receptor to the intracellular kinase domain that is linked to a sterile alpha motif (SAM) domain and PDZ-binding motif. Two tyrosine residues on the juxtamembrane region mediate autophosphorylation. Eph receptors bind to ephrin ligands via an extracellular Eph binding domain. Ephrin-A ligands are GPI-anchored to the plasma membrane and signal through co-receptors that have not yet been fully defined. Ephrin-B ligands are transmembrane and are linked to an intracellular PDZ-binding motif via a linker containing five tyrosine resides for autophosphorylation. **(B)** Dimerization of Eph receptors is regulated by various processes including SAM domain interactions, ligand clustering, and interactions between cysteine-rich regions and ephrin binding domains on neighboring receptors. Receptor dimerization mediates the formation of heterocomplexes that are required for signaling and are assembled via the Eph receptor PDZ-binding motif. Formation of the heterocomplex mediates bi-directional signaling in which numerous signaling pathways known to play a role in immune cell function can be activated through both ephrin “reverse” and Eph “forward” signaling. These signaling events include activation of Rho GTPases, MAP kinases, PI3 kinase, Src family kinases, Jak-STAT molecules, and RGS3 that has been shown to suppress G-protein coupled receptors including chemokine receptors. P, representative of tyrosine phosphorylation sites; GPCRs, G-protein coupled receptors; RGS3, regulator of G-protein signaling 3; Grb4, cytoplasmic protein NCK2; PI3K, phosphatidylinositol 3-kinase; AKT, protein kinase B; Cdc42, cell division control protein 42 homolog; Ras-GAP, Ras-GTPase-activating protein; Erk, extracellular signal-regulated kinases; Jak, Janus kinase; STAT, signal transducer and activator of transcription; IL, interleukin.

The Eph (erythropoietin-producing hepatocellular carcinoma) receptors represent the largest known family of receptor tyrosine kinases in mammals (1). These receptors are critical for a variety of normal cellular processes during development and are key mediators of adult tissue homeostasis (2–5). First discovered in a human carcinoma cell line (6), the Eph family of receptors is now known to include two classes of receptors that consist of 9 EphA members and 5 EphB members classified according to sequence homology (7) (Table 1). This group of receptors function through interactions with membrane-bound ephrin (Eph receptor-interacting protein) ligands to mediate changes in cellular shape, motility, migration, and proliferation (2, 4, 28, 29). The basic structures of Eph receptors and their ligands are shown in Figure 1A. All Eph receptors have a highly conserved overall structure with EphA and EphB receptors sharing the same structural features and domains. The primary sequence differences between EphA and EphB receptors reside in a region of the ligand binding domain determined to be a low-affinity ephrin binding site which is likely involved in determining ephrin subclass binding specificity (30). Given their high structural similarity, the differences in functional outcomes that result from activation of either EphA or EphB receptors can be primarily attributed to the spatial and temporal expression patterns of Eph receptors and ephrin ligands *in cis* on a cell and *in trans* on neighboring cells. In essence, activation of any given Eph receptor can have highly varied impacts on cellular processes depending on the cellular and microenvironmental context. EphA receptors bind promiscuously to ephrin-A ligands (five members) while EphB receptors bind promiscuously to ephrin-B ligands (three members) with some potential cross-talk between groups (31). In contrast to Eph receptors, the ephrin-A and ephrin-B ligand families have clear structural differences as ephrin-A ligands are tethered to the cell membrane through glycosylphosphatidylinositol (GPI) anchors while ephrin-B ligands have a short transmembrane domain and conserved cytoplasmic tail. Although it is widely accepted that clustering of membrane-bound Eph receptors and ephrin ligands is required to facilitate optimal signaling (32), research in cancer demonstrates that EphA2 expression on extracellular vesicles secreted from senescent cells can act on nearby ephrin-A1 expressing cancerous cells to contribute to proliferation (33). This indicates that cell-cell contact is not always necessary for the activation of downstream signaling upon Eph-ephrin contact. The complexity of interactions conveyed by this promiscuous binding leads to considerable diversity in functional output upon Eph-ephrin binding.

**Table 1:** List of Eph receptors expressed in both mice and humans along with potential binding partners and preferences for ephrin ligands.

**Receptor**	**Expressed in mice and humans?**	**Ligand interactions**
**EphA1** (6)	Yes	**Ephrin-A4** > Ephrin-A1 > Ephrin-A3 > Ephrin-A2, Ephrin-A5
**EphA2** (8)	Yes	**Ephrin-A1** > Ephrin-A5 > Ephrin-A4 > Ephrin-A3 > Ephrin-A2
**EphA3** (9)	Yes	**Ephrin-A5** > Ephrin-A4 > Ephrin-A2 > Ephrin-A3 > Ephrin-A1
**EphA4** (10)	Yes	**Ephrin-A4** > Ephrin-A5 > Ephrin-A1 > Ephrin-A2 > Ephrin-A3 ^*^Can also bind all Ephrin-B ligands (1–3)
**EphA5** (11)	Yes	**Ephrin-A5** > Ephrin-A3 > Ephrin-A4 > Ephrin-A2 > Ephrin-A1
**EphA6** (12)	Yes	**Ephrin-A5** > Ephrin-A4 > Ephrin-A2 > Ephrin-A1 > Ephrin-A3
**EphA7** (13)	Yes	**Ephrin-A5** > Ephrin-A3 > Ephrin-A4 > Ephrin-A1 > Ephrin-A2
**EphA8** (14)	Yes	**Ephrin-A4, Ephrin-A5** > Ephrin-A1 > Ephrin-A3 > Ephrin-A2
**EphA10** (inactive kinase) (15)	Yes	Ephrin-A1, Ephrin-A2, Ephrin-A3, Ephrin-A4, Ephrin-A5 (Binding affinities undetermined)
**EphB1** (16)	Yes	**Ephrin-B2** > Ephrin-B1 > Ephrin-B3 ^*^Can also bind Ephrin-A4
**EphB2** (14)	Yes	**Ephrin-B2** > Ephrin-B1 > Ephrin-B3 ^*^Can also bind all Ephrin-A ligands (1–5)
**EphB3** (17)	Yes	**Ephrin-B2** > Ephrin-B1 > Ephrin-B3 ^*^Can also bind Ephrin-A4
**EphB4** (18)	Yes	**Ephrin-B2** > Ephrin-B1 > Ephrin-B3 ^*^Can also bind Ephrin-A4
**EphB6** (inactive kinase) (19)	Yes	**Ephrin-B2** > Ephrin-B1 > Ephrin-B3 ^*^Can also bind Ephrin-A4
Additional references		(7, 15, 19–27)

A distinctive feature of Eph-ephrin interactions is the bidirectional signaling that occurs upon receptor-ligand binding and clustering which is termed forward signaling in Eph receptor-expressing cells and reverse signaling in ephrin ligand-expressing cells (34–36). Binding of Eph receptors to their ligands results in oligomerization and trans-phosphorylation leading to optimal kinase activity (37, 38). Importantly yet somewhat paradoxically, both cellular adhesion and repulsion can be consequences of Eph-ephrin binding between cells. High-affinity cell-cell binding events can lead to endocytosis of the receptor-ligand complex (39–41) or proteolytic cleavage of the ephrin extracellular domains (42–44) and cellular repulsion. On the other hand, Eph-ephrin adhesion is favored by reduced forward signaling (45, 46) and expression of Ephs and ephrins *in cis* (47, 48). There is also evidence that the fate decision between adhesion or repulsion can occur in a time-dependent manner where an initial adhesive event can later become a repulsive event (39, 42). The complexity provided by the signaling events downstream of Eph-ephrin binding allows for diverse functional consequences in a highly regulated and context-dependent manner, and examples of several potential signaling events that can occur upon Eph-ephrin clustering and ligation are shown in Figure 1B.

Expressed in most, if not all, adult tissues (2, 49), the Eph-ephrin signaling axis was initially most heavily studied for its complex role in embryonic and neural developmental processes such as cell segregation and migration, spatial organization of cell populations, tissue boundary formation, axonal guidance, and angiogenesis (50, 51). Also expressed on most cellular players of the immune system (Table 2), Eph-ephrin interactions have been implicated in various facets of immune surveillance including immune cell activation, migration, adhesion, and proliferation (93–95). In this review, we will discuss the emerging roles of Eph receptors and ephrin ligands in various aspects of immunity and disease pathogenesis as well as the implications of Eph receptors as future immunotherapy targets.

**Table 2:** Known protein expression profiles, functions, and disease contributions of Eph receptors and ephrin ligands on various immune cell subsets.

	**Immune cell subsets**				
	**Platelets**	**Monocytes and macrophages**	**Dendritic cells**	**B cells**	**T cells**
**Eph receptor protein expression**	EphA4, EphB1, EphB2	EphA2, EphA4, EphB2, EphB4	EphA2, EphB1, EphB2, EphB3	EphA3, EphA4, EphA7, EphA10, EphB2, EphB6	EphA1, EphA3, EphA4, EphB3, EphB4, EphB6
**Eph receptor functions**	Platelet activation, thrombus formation	Cell spreading and adhesion, extravasation	Cell organization and trafficking	B cell activation, proliferation, and antibody production	IL-21 production in germinal centers, TCR signaling, T cell activation, migration, functionality
**Cell-type specific disease relevance**	*EphB2* mutation associated with platelet dysfunction and/or recurrent bleeding in humans	Liver fibrosis, Arteriosclerosis	EphA2 serves as a herpesvirus entry receptor on DCs	EphA4 associated with B-cell lymphoma and post-transplant lymphoproliferative disorder	EphA3, EphB3 and EphB6 involved in T cell malignancies
**Ephrin ligand protein expression**	Ephrin-B1	Ephrin-A1, Ephrin-A2, Ephrin-A4, Ephrin-B2	Unknown	Ephrin-A1, Ephrin-A4, Ephrin-B1	Ephrin-A1, Ephrin-B1, Ephrin-B2, Ephrin-B3
**Ephrin ligand functions**	Thrombus stability	Cell-cell contact/adhesion	Unknown	Cell-cell contact/adhesion, germinal center interactions and organization	Thymocyte development, T cell differentiation, activation, costimulation, migration
**Cell-type specific disease relevance**	Unknown	Atherosclerotic plaque formation	Unknown	Relation to chronic lymphocytic leukemia progression	Contribution to rheumatoid arthritis pathogenesis, possible involvement in multiple sclerosis
**References**	(52–56)	(57–66)	(67–70)	(71–78)	(9, 78–92)

## Impact of Eph Receptor Expression on Stem Cell Fate

In order to understand how this unique family of receptors and ligands factors into immune system development and function, it is first necessary to review the effects of expression patterns of Eph receptors and their ligands on hematopoietic cells prior to divergence into different immune cell fates. There is evidence supporting a clear role for Eph receptors in cell fate decisions of hematopoietic progenitors prior to differentiation. EphB receptors in particular are important in the hematopoiesis of both red and white blood cells. Hematopoietic progenitor cells expressing EphB2 can be repulsed by bone marrow stromal cell-expressed Ephrin-B2, in turn mediating their subsequent differentiation into mature erythroid cells (96). Additionally, interactions between EphB4 and ephrin-B2 on bone marrow sinusoids and hematopoietic cells, respectively, aid in the mobilization of hematopoietic progenitor cells from the bone marrow (97). *In vitro*, ectopic EphB4 expression in hematopoietic cells promotes commitment to the megakaryocyte/erythroid lineage but not granulocytic or monocytic lineages (98). In the mouse small intestine, EphB2 is highly expressed on stem cells in crypts of villi while EphB3 is highly expressed on differentiated Paneth cells. The gradients of these receptors and their cognate ephrin-B1/B2 ligands tightly control cellular positioning and stem-cell differentiation and proliferation (99, 100). Various combinations and expression levels of Eph and ephrin family members have also been found on CD34^+^ stem cells in both the bone marrow (101) and peripheral blood (102). Since expression differs depending on hematopoietic stem cell location (bone marrow and blood) and other microenvironmental factors, expression patterns are likely important for both early development and later function of these cells prior to lineage commitment.

## Roles of Ephs and Ephrins in Immune Cell Activation

One of the initial processes in mounting an immune response is the activation of immune cells. There is evidence that Eph receptors and ephrin ligands may mediate immune cell activation. However, given the sparse number of reports in the literature it remains an open question of how signaling emanating from Eph-ephrin ligation influences activation and how this process is influenced by expression of these molecules *in cis* and *in trans* on different immune cell subsets. Furthermore, in the case of innate immune cells, initial recognition leads to activation that can be amplified through feedback loops once the adaptive immune response has been initiated. Below we outline what is currently known about the involvement of Eph receptors in activating both innate and adaptive immune cells.

### Innate Immune Cells

There are very few reports on the contribution of Eph receptors and ephrin ligands to the activation of innate immune cells. However, there is evidence suggesting a role for Eph receptors, specifically EphB2, in modulating dendritic cell (DC) responsiveness to toll-like receptor (TLR) ligation by pathogen-associated molecular patterns (69). Although not currently understood, it is possible that TLR signaling pathways intersect with EphB forward signaling events leading to a modulation of NFκB activation which is central to many immune cell activation pathways. Given the widespread expression of Eph receptors and TLRs on many innate immune cells (Table 2), it would be surprising if this is not a more widespread phenomenon although this remains to be tested in other innate immune cell types.

### B Cells

Eph receptors and ephrins have been identified on both human (72, 73, 103) and mouse (77) B cells. Eph receptors and ephrin ligands are also expressed differentially on naïve and activated B cells (73). This suggests that they may contribute to processes facilitating B cell activation as exemplified by naïve human B cells which upregulate EphB2 leading to increased proliferation and antibody production. In B cells, EphB2 has been demonstrated to be regulated by the microRNA miR-185 and its effects on B cell activation appear to occur at least in part through interactions between EphB2 and the Src-p65 and Notch1 signaling pathways (74). Thus, B cells may modulate expression of different Eph and/or ephrin members in order to facilitate their development, activation, differentiation, and functionality. Given the importance of cell-cell contact and localization to specific anatomical niches to the development, activation, and maturation of B cells, it seems likely that differential expression of Eph receptors and ephrin ligands could also contribute to B cell specialization into various canonical B cell fates.

### T Cells

Ephs and ephrins have been detected on human peripheral T cells with one study showing between 10% and 12% of CD4^+^ and CD8^+^ T cells expressing EphB6 (104). EphB receptors reported to be expressed on mouse naïve splenic CD4^+^ and CD8^+^ T cells include EphB1, EphB2, EphB3, and EphB6. Expression of all three ephrin-B ligands on T cells has also been demonstrated (79, 82, 105), but co-expression patterns of the ligands and receptors on T cells have not yet been determined. Given the fact that Eph receptors and ephrins are also present on antigen presenting cells such as DCs (67–70), expression of these molecules on T cells suggests a potential role in T cell activation and differentiation.

Multiple studies have demonstrated that all three ephrin-B ligands can influence cooperation between T cells, T cell co-stimulation, and enhancing signaling through the T cell receptor (TCR). EphB receptors and TCRs colocalize in signaling rafts on the surface of activated T cells. Additionally, ephrin-B1 mediated stimulation of T cells through EphB receptors increases both phosphorylation of LAT and activation of the signaling molecules p38 and p44/42 MAPK (79, 82) providing a mechanistic explanation for how Eph receptors and components of the TCR complex may interact. Specifically, EphB6 has been shown to have a critical role in T cell activation with EphB6 deficient mice displaying reduced activation, phosphorylation, and/or recruitment of the T cell signaling molecules ZAP-70, LAT, SLP-76, PLCγ1, and P44/42 MAPK (106). Administration of anti-EphB6 antibodies, which can cause EphB6 clustering and subsequent signaling on T cells, increases the response of mature T cells to weak TCR ligation as measured by canonical activation marker expression (CD25, CD69) and cytokine production (interferon (IFN)-γ, interleukin (IL)-6) as well as T cell proliferation (104). In support of its role in enhancing TCR signal strength, EphB6 cross-linking in a T cell line sensitive to strong TCR signaling leads to enhanced apoptosis (107) which supports the finding that strong Eph receptor activation can induce apoptotic pathways (36, 108). This is in contrast to EphA receptors which prevent apoptosis in thymocytes (109) and we speculate that this could also apply to peripheral mature T cells participating in an immune response.

More recently, it has been suggested that EphB-ephrin-B signaling may also provide negative feedback during T cell activation. Ephrin-B1 and ephrin-B2, but not ephrin-B3, co-stimulated T cells at low concentrations but inhibited T cell activation at higher concentrations. This inhibition at high ephrin-B1/B2 concentrations likely occurs through inducing recruitment of the SHP1 phosphatase in stimulated T cells resulting in reduced phosphorylation of the signaling molecules Lck, Erk, and Akt (88). Additional data shows that mixed lymphocyte reaction activated cells pulsed with EphB2-Fc or ephrin-B2-Fc recombinant chimeric proteins downregulate key activation molecules including IL-2, IFN-γ, tumor necrosis factor-α (TNF-α), and IL-17 (90). These data suggest that the context-dependent expression of a combination of EphB receptors and ephrin-B ligands on T cells may serve an immunomodulatory role in different microenvironments.

In addition to activation, it is possible that regulation of Eph receptors and ephrin ligands can influence T cell differentiation into various T cell subsets. EphA-ephrin-A interactions can preferentially skew the differentiation of activated human CD4^+^ T cells to a T helper (Th) type 1, rather than a Th2, phenotype upon cross-linking of ephrin-A ligands in the presence of α-CD3/α-CD28 activating antibodies via the suppression of IL-2 and IL-4 (81). This indicates that reverse signaling in ephrin-A ligand expressing T cells is able to intersect with other currently unidentified signaling pathways in order to suppress differentiation into a Th2 cell fate.

## Eph-ephrin Interactions Mediate Immune Cell Trafficking

**Figure 2:**
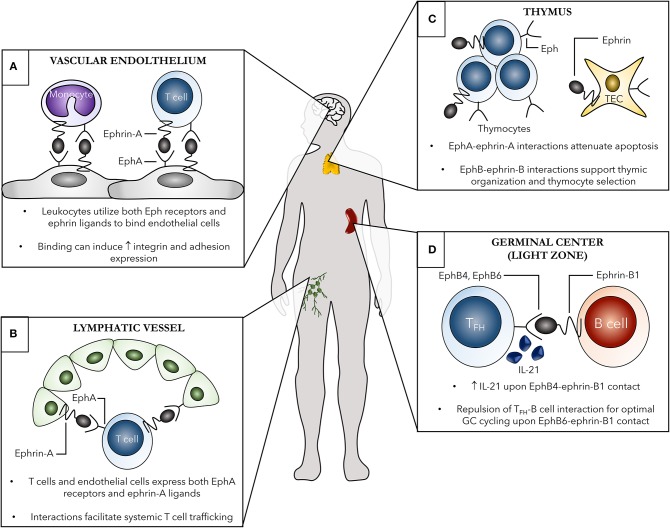
Examples of the contribution of Eph receptors and ephrin ligands to both localized and systemic immune cell trafficking. Several systemic **(A,B)** and localized **(C,D)** roles of Eph-ephrin interactions in immunity are shown. **(A)** Both leukocytes, such as monocytes and T cells, and vascular endothelial cells, shown here in the brain as an example, express various Eph receptors and ephrin ligands. Eph-ephrin interactions can aid in processes such as leukocyte chemotaxis, adhesion, and transmigration of the vascular endothelium. These binding events can subsequently induce increased expression of adhesion molecules and integrins leading to enhanced cell-cell contact. **(B)** EphA receptors, primarily EphA2, on high endothelial venules (HEVs) of lymph nodes can interact with ephrin-A ligand-expressing T cells to facilitate trafficking between the blood and lymph. Additionally, ephrin-A1 on HEVs can bind EphA receptors on circulating peripheral T cells leading to changes in actin polymerization in the T cell and initiating subsequent chemotaxis. **(C)** Thymic organization as well as thymocyte development are heavily dependent on Eph-ephrin interactions. The Eph B family members are particularly important in these processes, with both single- and double-positive thymocytes as well as thymic epithelial cells (TECs) expressing several EphB receptors and ephrin-B ligands to facilitate cellular organization of the thymus and thymocyte selection. **(D)** EphB-ephrin-B interactions are critical for optimal germinal center interactions between B cells and T follicular helper (T_FH_) cells. Ephrin-B1 marks a subpopulation of germinal center memory B cells and binding to EphB4 and EphB6 on T_FH_ cells induces IL-21 production from the T_FH_ cells and repulsion, respectively, both required for optimal germinal center B cell cycling.

An emerging role for Eph receptors and ephrin ligands in immune cell trafficking has been a focus of recent research. Cell migration is a fundamental process required for optimal immune system functioning. Migration occurs both locally, where cells are required to move within lymphoid organs such as the spleen in order to interact with other immune cells, and systemically during routine immune surveillance and response to damage or infection. Several key events in local and systemic immune cell trafficking that involve Eph-ephrin interactions in both steady and activated states of the immune system are shown in Figure 2 and discussed in detail below.

### Localized Immune Cell Migration Within Lymphoid Organs

#### Thymic Movement of T Cells During Development

It is not surprising that members of the Eph family would mediate a process such as T cell maturation given their well-established roles in tissue organization and cell migration in numerous organ systems. During T cell maturation, immature thymocytes undergo multiple steps of selection in the thymus requiring extended cell-cell contact with various cell types such as thymic stromal cells. It is quite possible that Eph-ephrin interactions may mediate adhesion and aid in the movement of maturing T cells through different thymic compartments during this process. Indeed, the expression of nearly all Eph receptors and ephrin ligands, with the exception of a few, has been detected in the thymus (19, 110). In the fetal thymus, EphB2 and EphB3 appear to be crucial for the successful development of thymic epithelial cells (111). Furthermore, EphB2 is implicated in mediating the colonization of T cell progenitor cells during fetal thymus colonization (112). As such, the presence of EphB2 and ephrin-B1/B2 ligands on thymocytes and thymic epithelial cells is essential for the correct organization of the thymic medulla (113). EphB6 is highly expressed in thymocytes and in mice between 50% and 70% of T cells in the thymus express EphB6 including CD4^+^CD8^+^ double positive as well as CD4^+^ and CD8^+^ single positive T cells (106). However, only around 8–17% of peripheral mature CD4^+^ and CD8^+^ T cells in mice are EphB6^+^ (106) indicating a potential role for EphB6 and its cognate ligand in mediating T cell retention in the thymus during development.

Although there have not been a large number of studies that address the role of Eph-ephrin interactions in T cell migration and adhesion within the thymus, such interactions appear to be critical for modulating apoptosis of thymocytes and are therefore likely critical in T cell selection processes. Distinct, yet overlapping, expression patterns of EphA receptors and ephrin-A ligands are observed in the rat thymus, and disruption of these interactions in a thymic culture model with EphA-Fc or ephrin-A-Fc recombinant chimeric proteins leads to an increase in apoptosis of double positive CD4^+^CD8^+^ T cells (114). This is in contrast to one study that showed ligation of EphA receptors with ephrin-A1 ligand can prevent apoptosis in thymocytes (109) but in agreement with a mouse study that used recombinant EphB2-Fc or ephrin-B1-Fc in thymic organ cultures *in vitro* to show reduced numbers of thymocytes via increased apoptosis (115). An overwhelming number of *in vivo* mouse models involving both Eph receptor subfamilies support the conclusion that Eph-ephrin interactions prevent apoptosis of thymocytes. Mice with selective T cell deficiency of ephrin-B1 and ephrin-B2 have significantly reduced numbers of double positive and single positive T cells in the thymus compared with intact littermate control mice (116). However, the lack of an effect in mice with a selective T cell deficiency in ephrin-B2 only (87) demonstrates the redundancy of this family of molecules as well as the ability of these receptors and ligands to potentially compensate for one another. Reduced thymic cellularity is also observed in the absence of EphB2, EphB3 (117), and EphA4 (118) in mice again suggesting a critical role for Eph receptors in preventing apoptosis of thymocytes during T cell thymic maturation.

#### Movement of Immune Cells in the Context of Germinal Centers

Germinal centers (GCs) are key immunological structures in secondary lymphoid organs that form to facilitate the interaction between T follicular helper (Tfh) cells and activated B cells and to aid in the development of a robust humoral immune response. During an early GC reaction, activated Tfh cells interact with their cognate antigen-specific B cells to promote B cell proliferation and differentiation into plasma cells and memory B cells (119). With T and B cells activated in different zones within the GC, this cellular interaction requires movement of T cells into the B cell zone as induced by the chemokine CXCL13 (previously known as B-cell attracting chemokine-1) along with subsequent exit of antibody-secreting plasma cells and memory B cells from the GC. In order for these extensive cell cycling and cell-cell contact events to successfully occur in the GC, both attractive and repulsive events between cells are required.

One of the more recently discovered immunological roles of the Eph-ephrin signaling system involves the generation of GC B cell responses. Ephrin-B1, a ligand that can bind to several EphB receptors, was recently shown to be a marker of mature GC B cells and may specifically differentiate early GC memory precursor B cells from other subsets of GC B cells (77). The development of an optimal humoral immune response requires specific temporal interactions to occur between Tfh cells and GC B cells. Ephrin-B1 has been shown to be involved in the localized interaction between B cells and Tfh cells in the GC microenvironment (78). Specifically, GC B cell-expressed ephrin-B1 can inhibit recruitment and retention of Tfh cells in the GC and is required for GC B cells to induce optimal levels of IL-21 from Tfh cells via EphB4 forward signaling (78). Given the key role of IL-21 in plasma cell formation and affinity maturation, GC B cell-expressed ephrin-B1 is therefore a key molecule required for the optimal functioning of GC interactions as a whole.

### Migration of Immune Cells Systemically

Directional trafficking of immune cells throughout the body is driven in part by chemokine receptors expressed on immune cells that respond to chemokines secreted by distal cells. The process of systemic cell migration is facilitated by adhesion molecules such as integrins expressed on vascular endothelial cells and their ligands expressed on trafficking immune cells. Like integrins, Ephs, and ephrins are also expressed in the vasculature throughout the body (49, 120, 121). Given the widespread expression of Eph receptors and ephrin ligands on immune cells, the Eph-ephrin family of molecules may also be an important family of molecules facilitating trafficking of immune cells to the site of damage and inflammation.

The Eph-ephrin system has been shown to participate in multiple steps of monocyte trafficking including chemotaxis, adhesion (60), and vascular endothelial transmigration (57). In particular, expression of the receptors and ligands EphA2, EphA4, ephrin-A1, ephrin-A2, and ephrin-A4 is upregulated on mouse and human classical monocyte subsets at both the RNA and protein level, and these molecules can contribute to adhesion of monocytes to integrin-coated surfaces (65, 66, 122). One example of how Eph receptor signaling may mediate monocyte retention at the site of inflammation involves the interaction between ephrin-A1 on monocytes and EphA4 on endothelial cells. Upon endothelial EphA4 activation, the RhoA signaling pathway is activated leading to increased actin filament polymerization and subsequently enhanced monocyte-endothelial cell adhesion (60). Overall, the signaling events downstream of Eph-ephrin activation are likely to participate in crosstalk with integrin molecular pathways and have been hypothesized to facilitate adhesion of monocytes to other Eph or ephrin-expressing cells.

Human DCs are also known to express members of the EphA (EphA2, EphA4, EphA7) and EphB (EphB1, EphB2, EphB3, EphB6) subfamilies (68, 69). Similar to monocytes, Eph receptors could potentially contribute to DC trafficking and adhesion by allowing for localization to sites of damage or infection in the body with some evidence suggesting that crosstalk between Eph receptors and integrins, particularly β1 integrin, may facilitate DC adhesion (68). Interestingly, different subsets of DCs such as Langerhans cells (123), interstitial DCs, and plasmacytoid DCs (67) have unique patterns of Eph receptor expression. Although the reasons for these unique expression patterns remain unknown, it is possible that Eph receptors may contribute to the locational and functional specificity observed in these different DC subsets.

In the adaptive arm of the immune system, members of the EphA-ephrin-A family have been associated with B and T cell trafficking in several studies. The expression of EphA2 on high endothelial venules (HEVs) in human lymph nodes (124) suggests a role in immune cell trafficking. In support of this data, initiation of ephrin-A reverse signaling on T cells alters T cell trafficking by directing the T cells to enter lymph nodes upon injection into recipient mice (84). Although the specific member(s) of the ephrin-A ligand family important for this phenomenon is currently unknown, EphA2 has been shown to be important in mediating T cell trafficking between the blood and lymph nodes through interactions with ephrin-A4 on peripheral T cells (124). Along the same lines, ephrin-A1 is also expressed on HEV endothelial cells, and engagement of EphA receptors on the surface of both CD8^+^ and CD4^+^ T cells directly stimulates chemotaxis through effects of this ligation on actin polymerization (125). Specifically, the migration that is induced upon stimulation of EphA receptors on T cells with ephrin-A ligands involves activation of a variety of signaling molecules including Lck, Pyk2, PI3K, Vav1, and Rho GTPase (126). More recently, it has also been shown that activation of EphA2 on endothelial cells with ephrin-A1 leads to NFAT activation and subsequent upregulation of vascular cellular adhesion molecule 1 (VCAM-1) which aids in leukocyte recruitment by facilitating cellular adhesion (127). Collectively, these studies indicate a key role for Eph molecules, particularly EphA2, in optimal adaptive immune cell trafficking to sites of inflammation.

Interestingly, the expression of ephrin-A ligands on the various subsets of CD4^+^ and CD8^+^ T cells differs (85) and thus EphA-ephrin-A expression patterns may contribute to the differing migratory potential observed in naïve, effector, and memory T cells. Expression of ephrin-B ligands has also been implicated in T cell trafficking to inflamed paws during a collagen-induced arthritis mouse model (91) as well as to the central nervous system in the experimental autoimmune encephalomyelitis (EAE) mouse model (92) underlining a role for both subfamilies in T cell trafficking to distal sites of inflammation in the body. Interplay between Eph-ephrin interactions and chemokines has yet to be well-studied but T cell chemotaxis in response to the chemokines stromal cell derived factor-1α (SDF-1α, also known as CXCL12) and macrophage inflammatory protein-3β (MIP-3β, also known as CCL19) can also be modulated by both ephrin-A and ephrin-B ligands (128) implicating both subfamilies in T cell trafficking in response to chemokine gradients.

## Involvement of Eph Receptors and Ephrin Ligands in Disease Pathogenesis

The ubiquitous expression profile of Ephs and ephrins throughout the human body makes them plausible candidates for mediating a variety of immunological processes. However, the widespread expression pattern along with the characteristic tyrosine kinase activity associated with Eph receptors also renders them highly likely to contribute to certain pathological conditions. Evolving research implicates various members of this family in a growing number of immune-mediated pathological conditions (59, 129, 130) as well as the pathogenesis of various diseases (3, 131–133). Thus, Eph receptors and ephrin ligands have become attractive therapeutic targets for several diseases including cancer, neurological disorders, and infectious diseases (76, 131, 134–140). In the remaining sections, we discuss what is currently known about the involvement of the Eph-ephrin family in both non-infectious and infectious diseases to convey the complexity of how these molecules contribute to various diseases.

### Cancer

The first Eph receptor was identified in 1987 (6) from a human carcinoma cell line in a screen for oncogenic tyrosine kinases. It is therefore not surprising that a strong link between Eph receptors and cancer has emerged in the years since their discovery. Various Eph receptors and ephrin ligands are expressed in cancer cells as well as cells in the tumor microenvironment allowing for cell-cell communication within these compartments (131, 141). In the tumor microenvironment, upregulation of ephrin-A2, ephrin-A3, EphB2, and EphB4, to name a few, on vascular cells in response to tumor factors has been the most well-studied (49, 142). EphA2 and EphB4 are upregulated in many types of cancers and are associated with increases in cancer malignancy and poor prognosis (143–146). Expression of several EphB receptors has also been inversely correlated with colorectal cancer where decreased expression is associated with increased malignancy (147).

As many Eph receptors and ephrin ligands are expressed on T and B cells (Table 2) and can play critical roles in cell development, differentiation, activation, and proliferation, it is predictable that aberrant expression or activation of these molecules on adaptive immune cells could contribute to hematologic malignancies. EphA3 has been of particular interest to the cancer research field as it was originally identified in an acute lymphoblastic leukemia cell line and expression can be detected in many T cell lymphomas but not generally in T cells from healthy individuals (9, 71, 80, 148). Given its unique expression on malignant T cells, EphA3 has strong potential to serve as a therapeutic target for EphA3^+^ T cell lymphomas with minimal detrimental effects on healthy T cells. Indeed, an anti-EphA3 monoclonal antibody has already been the subject of a Phase I clinical trial in patients with refractory hematologic malignancies (149). In addition to its role in T cell lymphomas, EphA3 also plays a role in multiple myeloma angiogenesis (150) suggesting that an effective anti-EphA3 therapy could be potentially utilized for treatment of both T and B cell malignancies. Along with EphA3, expression of the receptors EphA2, EphB3, and EphB6 has also been identified in many malignant T lymphocytes (86, 151). The potential involvement of these receptors in promoting survival of malignant immune cells suggests that they may also hold promise as future therapeutic targets. However, Eph receptors may also be involved in lymphoma suppression as has been shown in the case of a soluble form of EphA7 (152) and for EphB4 (153). This indicates that great care must be taken in designing future anti-Eph therapies to ensure specificity toward targeting particular Eph receptors of interest that may contribute to malignancies while avoiding targeting those that may benefit the host anti-tumor immune response.

Paradoxically, both an increase and a decrease in Eph receptor expression has been associated with cancer progression consistent with the functional complexity of interactions between different Eph-ephrin family members. For the most part, the roles for Eph receptors in malignancies have been investigated from a cellular biology perspective. For example, Eph receptor forward signaling can inhibit cancer cell migration, proliferation, and survival as well as tumor growth in mice (154). Angiogenesis is essential for both tumor growth and metastasis and some evidence suggests that interactions between EphA2 and ephrin-A1 (50, 144) as well as EphB4 and ephrin-B2 (50, 144) on tumor cells and vascular cells can lead to increased angiogenesis and tumor vascularization. Several recent studies have aimed to alter or redirect T cells to target Eph receptors on cancerous cells using unique approaches including the generation of chimeric antigen receptor (CAR) T cells in which the T cell receptor recognizes EphA2 (155–157). A second approach which involves administering a bi-specific antibody that recognizes EphA10 expressed on breast cancer cells as well as CD3 expressed on T cells aims to redirect cytotoxic CD8^+^ T cells to attack malignant EphA2^+^ cells (158). However, these studies have not comprehensively addressed the potentially negative effects that Eph-ephrin expression on T cells may have on the target cells, which often will also express the same receptors and their ligands. While the contribution of Eph-ephrin expression on cancerous cells to disease progression has been thoroughly investigated, the crucial role of the immune response in the recognition and containment of malignant cells is often overlooked. Given the nearly ubiquitous expression of Eph receptors on immune cells, the function that these receptors play in cancer immunology must also be understood in order to rationally design Eph-based anti-cancer therapeutics that incorporate the contributions of Eph receptors to the immune control of cancers.

### Atherosclerosis

Atherosclerosis is a condition characterized by the hardening and narrowing of the arteries which can impede blood flow and lead to heart attack and stroke. Damage to the vascular endothelium triggers the process of atherosclerosis, and activation of platelets by subendothelial matrix-derived molecules such as collagen and fibronectin triggers the adhesion of platelets onto the endothelium. This, in turn, initiates the formation of plaques that harbor immune cells such as monocytes and macrophages which have migrated to the plaque in response to chemotactic signals (159).

Platelets, anuclear cell fragments derived from megakaryocytes, are components of the immune system capable of exerting effects on both the innate and adaptive branches. Human platelets express several members of the Eph-ephrin family including EphA4, EphB1, EphB2, and ephrin-B1. Forward signaling through the EphB2 receptor on platelets is associated with both thrombus formation and platelet activation in the absence of ligand contact (55). This suggests an inherent role of the Eph cytoplasmic signaling domain in contributing to platelet function. The key contribution of EphB2 to platelet activation and optimal functioning is also supported by the association between mutations in the human *EphB2* gene and platelet dysfunction (56). Ligation of this family of molecules on platelets may contribute to granule secretion along with adhesive interactions between platelets. Subsequent platelet aggregation and thrombus formation can then follow mediated by α_IIb_β_3_ integrin (52, 54, 160). Platelet granule release upon platelet activation is a highly inflammatory event. As such, Eph-mediated platelet activation is likely a key event mediating the immunopathogenic aspects of atherosclerosis.

In addition to platelets, other cells of the immune system can influence and enhance the inflammatory milieu of atherosclerotic plaques via Eph-ephrin interactions. Several studies have demonstrated a positive correlation between expression of EphB2, EphB4, ephrin-B1, and ephrin-B2 and the macrophage content of atherosclerotic plaques. These particular members of the Eph-ephrin family can contribute to the recruitment (161) and proinflammatory activation (58) of monocytes. Inflammation is likely further compounded by a suppression of localized chemotactic gradients by monocytes and/or macrophages leading to retention of these cells within lesions and augmentation of inflammatory responses (162). More work is needed to define precisely how Eph-ephrin interactions contribute to the pathogenesis of atherosclerosis. However, the abilities of these molecules to modulate the activation and chemotaxis of monocytes and macrophages, key events mediating plaque formation and growth, suggest a potential therapeutic avenue for this disease.

### Fibrosis

Fibrosis describes the scarring of tissue that occurs through excess production of extracellular matrix proteins in an attempt to repair damaged tissue. While the causes of fibrosis can be highly varied, the general process is thought to result from chronic inflammation leading to the activation of myofibroblasts that produce molecules such as collagen and glycosaminoglycans in response to immune mediators such as transforming growth factor (TGF)-β (163). Recently, Eph receptors and their ligands have been implicated in the development of organ fibrosis. EphB2 has been identified as a pro-fibrogenic molecule that is essential for the development of liver fibrosis from both infectious and non-infectious etiologies (63). By mediating trafficking of ephrin-B-expressing macrophages to the liver, EphB2 can promote inflammatory signaling pathways that stimulate the differentiation of hepatic stellate cells into fibrogenic myofibroblasts. In a separate model of lung fibrosis, cleavage of the ligand ephrin-B2 on fibroblasts by the disintegrin and metalloproteinase domain-containing protein ADAM10 leads to increased fibroblast activation and subsequently increased skin and lung fibrosis (164). It is anticipated that similar processes involving Eph-ephrin interactions may underlie other fibrotic conditions such as systemic scleroderma.

### Diseases of the Central Nervous System

Eph-ephrin interactions play a key role in neurological development, and neurological disorders can stem from dysfunctional Eph-ephrin interactions. Upregulation of EphA4 expression has been consistently observed after traumatic brain injury in both primates (165) and humans (166). Similarly, EphA4, along with several other Eph receptors, appears to inhibit neuronal regrowth and recovery after spinal cord injury in mice (167).

Multiple sclerosis (MS) is a disease in which the nerve cells of the brain become demyelinated and exposed rendering them unable to communicate efficiently. This leads to a variety of physical symptoms such as muscle weakness and poor coordination. It is the most common immune-mediated disease of the central nervous system (168) in which auto-reactive CD4^+^ T cells play a central role in contributing to the induction of inflammation and lesion formation in the brain and spinal cord. Increased expression of ephrin-A1, EphA3, EphA4, and EphA7 has been observed in axons of multiple sclerosis lesions (169). Ephrin-B1 and ephrin-B2 were also shown recently to be involved in T cell migration to the central nervous system in both the mouse model of EAE and in human MS (92) which indicates the potential importance of these molecules in mediating this disease. However, there remains a significant amount of knowledge to be gained before the full contribution of the Eph-ephrin molecules to MS can be fully understood.

Other diseases of the central nervous system that may involve Eph-ephrin interactions include Parkinson's disease (170) and Alzheimer's disease (171). Although studies thus far have only identified correlations between Eph expression and disease severity, the known roles for Ephs and ephrins in the immune system outlined in this review make these correlations worthy of further investigation.

### Infectious Diseases

Given the central role of the immune system in defense against infectious pathogens, there is a surprising lack of reports regarding the involvement of Eph receptors in this regard although this has become a greater focus of research in recent years. A clear role for Ephs as viral entry receptors has been shown for Nipah and Hendra viruses (ephrin-B2 and ephrin-B3) (172, 173), Kaposi's sarcoma-associated herpesvirus (EphA2) (70, 174), and Epstein-Barr virus (EphA4 and EphA2) (76, 138, 140). Similarly, it was also recently described that the sporozoite stage of the *Plasmodium* parasite, the causative agent of malaria, engages EphA2 on liver hepatocytes in order to establish a productive infection (135) although additional entry receptors may be involved in this process as well (175). EphA2 is also used by the fungal pathogen *Cryptococcus neoformans* to traverse the blood-brain barrier in order to gain entry into the brain (137).

In addition to facilitating entry, it is likely that the ubiquitous expression of Eph receptors and ephrin ligands on the majority of cells in the immune system facilitates immune defense, although reports in support of this idea are currently sparse. One of the challenges in the study of Eph-ephrin molecules is the redundancy that can occur with respect to binding of Eph receptors to multiple ephrin ligands. Although one study demonstrates no effect of B cell-specific ephrin-B1 ligand deficiency on GC formation, plasmablast production, or B cell class-switching in mice after infection with either influenza virus or acute lymphocytic choriomeningitis virus (LCMV Armstrong) (77), compensatory activities of other ephrin-B ligands may mask a potential role of the ephrin-B ligands in general. For example, a T cell-specific dual deletion of both ephrin-B1 and ephrin-B2 does in fact lead to a defective immune response against the LCMV virus (116). One additional study demonstrates that *Mycobacterium tuberculosis* can manipulate EphA2 and ephrin-A1 expression in order to support granuloma formation (176) which, in turn, aids in bacterial immune evasion. These reports suggest that these molecules do play a role in the activation and regulation of immune responses against pathogens further highlighting the importance of this consideration in future therapeutic designs.

## Concluding Remarks and Future Perspectives

The study of Eph receptors and ephrin ligands in immunology is an expanding and exciting area of research. Given that the association of this family of molecules with the immune system has only emerged in the past few decades, it is highly likely that many of the roles for the Eph-ephrin cell-cell communication system in immunity have yet to be revealed.

Ephs and ephrins represent promising therapeutic targets for immune manipulation. Although some of the challenges to this approach include the nearly ubiquitous expression of Ephs and ephrins and the often dichotomous consequences of Eph receptor signaling in different contexts, the high evolutionary conservation of this family of molecules increases the chances that Eph-based therapeutics developed and tested in animal models have the potential to be highly translatable to humans. Indeed, there are several Eph-ephrin targeting strategies currently progressing through clinical trials (134, 136, 177) that include blocking receptor-ligand binding as well as downstream signaling using a variety of therapeutic compounds. The potential repurposing of these drugs for new pathological indications is also attractive. Emerging roles for Eph-ephrin signaling in normal immune system functionality along with their contributions to disease pathogenesis guarantee an exciting new chapter in Eph biology and drug discovery.

## Author Contributions

This review was conceptualized by TD and TL. The initial draft was written by TD with subsequent drafts edited and approved for publication by both TD and TL.

### Conflict of Interest Statement

The authors declare that the research was conducted in the absence of any commercial or financial relationships that could be construed as a potential conflict of interest.
